# Potent and Selective Inhibition of CYP1A2 Enzyme by Obtusifolin and Its Chemopreventive Effects

**DOI:** 10.3390/pharmaceutics14122683

**Published:** 2022-12-01

**Authors:** Eun-Ji Park, Keunwan Park, Prasannavenkatesh Durai, Ki-Young Kim, So-Young Park, Jaeyoung Kwon, Hee Ju Lee, Cheol-Ho Pan, Kwang-Hyeon Liu

**Affiliations:** 1 BK21 FOUR KNU Community-Based Intelligent Novel Drug Discovery Education Unit, College of Pharmacy and Research Institute of Pharmaceutical Sciences, Kyungpook National University, Daegu 41566, Republic of Korea; 2 Natural Product Informatics Research Center, KIST Gangneung Institute of Natural Products, Gangneung 25451, Republic of Korea; 3 Mass Spectrometry Based Convergence Research Institute, Kyungpook National University, Daegu 41566, Republic of Korea

**Keywords:** *Cassiae* semen, chemoprevention, CYP1A2, inhibition, obtusifolin

## Abstract

Obtusifolin, a major anthraquinone component present in the seeds of *Cassia tora*, exhibits several biological activities, including the amelioration of memory impairment, prevention of breast cancer metastasis, and reduction of cartilage damage in osteoarthritis. We aimed to evaluate the inhibitory effects of obtusifolin and its analogs on CYP1A enzymes, which are responsible for activating procarcinogens, and investigate its inhibitory mechanism and chemopreventive effects. P450-selective substrates were incubated with human liver microsomes (HLMs) or recombinant CYP1A1 and CYP1A2 in the presence of obtusifolin and its four analogs. After incubation, the samples were analyzed using liquid chromatography-tandem mass spectrometry. Molecular docking simulations were performed using the crystal structure of CYP1A2 to identify the critical interactions between anthraquinones and human CYP1A2. Obtusifolin potently and selectively inhibited CYP1A2-mediated phenacetin *O*-deethylation (POD) with a *K*_i_ value of 0.031 µM in a competitive inhibitory manner in HLMs, whereas it exhibited negligible inhibitory effect against other P450s (IC_50_ > 28.6 µM). Obtusifolin also inhibited CYP1A1- and CYP1A2-mediated POD and ethoxyresorufin *O*-deethylation with IC_50_ values of <0.57 µM when using recombinant enzymes. Our molecular docking models suggested that the high CYP1A2 inhibitory activity of obtusifolin may be attributed to the combination of hydrophobic interactions and hydrogen bonding. This is the first report of selective and potent inhibitory effects of obtusifolin against CYP1A, indicating their potential chemopreventive effects.

## 1. Introduction

*Cassiae* semen (the seeds of *Cassia obtusifolia* L. and *Cassia tora* L.) is used as a traditional herbal medicine to protect the liver, improve vision, and treat photophobia in most regions of Asia, including Korea, China, and Japan [1]. Numerous phytochemicals, including anthraquinones, naphthopyrones, phytosterols, and volatile oils, have been isolated from *Cassiae* semen [2]. Among these anthraquinones (chrysophanol, emodin, obtusifolin, obtusin, and physcion) and naphthopyrones (rubrofusarin) are the primary bioactive substances present in *Cassiae* semen [1,3,4]. Obtusifolin, the most abundant anthraquinoid in the seeds of *Cassia tora* [3], ameliorates memory impairment [5], prevents breast cancer metastasis [6], and reduces cartilage damage in osteoarthritis [7].

Anthraquinones are a class of natural phenolic compounds with pharmacological properties, including anticancer, anti-inflammatory, antimicrobial, and antioxidant activities [8]. Several anthraquinoids demonstrated CYP1A2 inhibitory potential. Emodin, purpurin, and rubiadin selectively inhibited CYP1A2-mediated phenacetin-*O*-deethylation (POD) activity with IC_50_ values of 0.5, 2.3, and 1.3 µM, respectively, in human liver microsomes (HLMs); at the same time, these exhibited negligible inhibition of CYP2C9, CYP2C19, CYP2D6, and CYP3A (IC_50_ > 9.6 µM) [8]. However, the effects of obtusifolin, chrysophanol, and physcion, the major components of *Cassiae* semen, on cytochrome P450 (P450) have not been investigated in HLMs to date.

In humans, the CYP1A family is composed of two major isoforms, namely CYP1A1 and CYP1A2 [9]. CYP1A enzymes are preliminarily regulated by their aromatic hydrocarbon receptors and exhibit aryl hydrocarbon hydroxylation activity [10]. They are critical in activating procarcinogens such as aflatoxins and polycyclic aromatic hydrocarbons. Reactive carcinogenic intermediates (aflatoxin B1-exo-8,9-epoxide or benzo[a]pyrene-7,8-epoxide) produced by the upon the action of CYP1A enzymes ultimately form adducts with DNA [11,12]. Previous reports suggest that CYP1A contributes to tumor formation. Therefore, selective and robust inhibitors of CYP1A enzymes may act as effective chemopreventive agents for cancer treatment. Notably, resveratrol and sulforaphane have been considered as potent chemopreventive agents because they directly inhibit CYP1A enzymes [13,14].

CYP1A inhibitors share planar and compact structure with hydrophobic aromatic rings. For example, α-naphthoflavone (ANF), emodin, and 7-ethynylcoumarins, which are planar aromatic compounds, strongly inhibited CYP1A1 and CYP1A2 activities with IC_50_ values of <0.50 µM [15,16,17]. Obtusifolin, an anthraquinone isolated from *Cassiae* seeds, is also a planar aromatic compound; however, enzyme inhibition studies assessing the effect of obtusifolin on CYP1A activity have not been attempted.

Therefore, we evaluated the CYP1A2 inhibitory potential of obtusifolin, chrysophanol, emodin, physcion, and rubrofusarin, which are the major components of *Cassiae* semen, in HLMs. The difference in CYP1A2 inhibitory ability of the five phytochemicals was analyzed based on the interaction between the active site structure of the CYP1A2 enzyme and phytochemicals. An inhibition mechanism was identified for obtusifolin, which exhibited the most substantial inhibition of CYP1A2 activity; the inhibitory potential of obtusifolin against other nine P450 isoforms was also investigated to elucidate its selectivity in terms of CYP1A2 inhibition. In conclusion, we have demonstrated that obtusifolin acts as a selective CYP1A1 and CYP1A2 inhibitor in HLMs and also inhibits the activity of recombinant CYP1A enzymes, and is a promising chemopreventive candidate.

## 2. Materials and Methods

### 2.1. Chemicals and Enzymes

**Figure 1:**
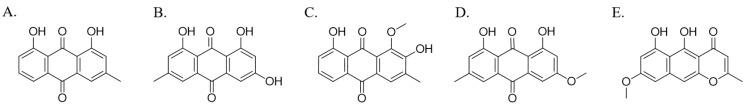
Chemical structures of four anthraquinones and one naphthopyrone from *Cassiae* semen: chrysophanol (**A**), emodin (**B**), obtusifolin (**C**), physcion (**D**), and rubrofusarin (**E**).

The following chemicals were obtained from Sigma-Aldrich (St. Louis, MO, USA): amodiaquine, bupropion, chlorzoxazone, dextromethorphan, 7-ethoxyresorufin, glucose-6-phosphate (G6P), G6P dehydrogenase (G6PDH), β-nicotinamide adenine dinucleotide phosphate (NADP^+^), omeprazole, phenacetin, and trimipramine. Coumarin, midazolam, and tolbutamide were obtained from Toronto Research Chemicals (Toronto, ON, Canada). We purchased resorufin from Tokyo Chemical Industry (Tokyo, Japan). Pooled human liver microsomes (pHLMs, XTreme 200) were obtained from XenoTech (Kansas City, KS, USA), and human recombinant CYP1A1 (rCYP1A1) and CYP1A2 (rCYP1A2) isoforms were obtained from SPMED (Busan, Republic of Korea). Chrysophanol (95.0% purity), emodin (94.3% purity), obtusifolin (95.4% purity), physcion (95.0% purity), and rubrofusarin (90.0% purity) (Figure 1) were kindly provided by the Korea Institute of Science and Technology (Gangneung, Republic of Korea).

### 2.2. CYP1A2 Activity Assays

The inhibitory potential of five major bioactive compounds from *Cassiae* semen against CYP1A2-mediated phenacetin *O*-deethylase (POD) activity was evaluated using previously developed methods with minor modifications [18]. Briefly, the compounds were dissolved in methanol. The incubation mixtures containing pHLMs (0.25 mg/mL), phenacetin (100 µM), and inhibitor (0, 0.5, 2, 5, 20, or 50 µM) were pre-incubated at 37 °C for 5 min. An NADPH-generating system (NGS, 1.3 mM β-NADP^+^, 3.3 mM G6P, 1.0 U/mL G6PDH, and 3.3 mM magnesium chloride) was introduced after pre-incubation to initiate the reaction, and further incubated for 15 min. The reaction was quenched by the addition of 50 µL ice-cold acetonitrile containing internal standard (IS; 7nM trimipramine). Aliquots of supernatants were analyzed by liquid chromatography–tandem mass spectrometry (LC-MS/MS, LCMS 8060 system, Shimadzu, Kyoto, Japan). All microsomal incubations were repeated three times.

### 2.3. Molecular Docking Simulation

The X-ray crystal structure of α-naphthoflavone bound to CYP1A2 was retrieved from the protein data bank (PDB) under accession number 2HI4 [19]. The protein structure was cleared of all water molecules except those at the active site. Building missing loops, energy minimization, and protonation in Discovery Studio Client (DSC) v19.1 produced the final protein structure with heme; this was used for further molecular docking analysis. The anthraquinone structures were drawn using ChemDraw 20.1.1. These structures were converted to a 3D format in DSC 19.1 by energy minimization after adding hydrogen atoms. The grid center was derived using α-naphthoflavone in the crystal structure, and 1000 docking runs were performed for obtusifolin with SMINA, a fork of AutoDock Vina using Gnina 1.0 [20]. The redundant final poses were filtered using root-mean-square deviation (rmsd) values. The other four active compounds of *Cassiae* semen (three anthraquinones and one naphthopyrone) were also docked by aligning to the obtusifolin-binding pose (“Align to substructure” option in DSC v19.1) to understand the structural and molecular interactions responsible for the variations in their inhibitory activity against CYP1A2. For the docked CYP1A2-ligand complexes, AutoDock VINA scores were calculated using the “score_only” option in Gnina 1.0. The molecular docking procedure was validated by re-docking α-naphthoflavone in the crystal structure; the rmsd value of the docked α-naphthoflavone and its crystal form was 0.3 Å, showing a high agreement (Figure S2). The figures depicting the protein–ligand interactions were generated using DSC v19.1.

### 2.4. Time-Dependent Inhibition Assays

The IC_50_ shift approach was applied to assess the time-dependent inhibition of CYP1A2 by obtusifolin [18]. For 30 min, obtusifolin was pre-incubated with pHLMs in the presence of NGS. The reaction was started with 100 µM phenacetin and then incubated for 15 min. Following reaction termination and centrifugation, aliquots of the supernatants were subjected to LC-MS/MS analysis.

### 2.5. Kinetic Characterization of CYP1A2 Inhibition by Obtusifolin in Human Liver Microsomes and Recombinant CYP1A2

The mechanism by which obtusifolin and α-naphthoflavone inhibit CYP1A2-mediated POD activity was studied in pHLMs or recombinant CYP1A2 (10 pmol/mL). Obtusifolin (0, 0.02, 0.05, 0.2, and 0.5 µM) or α-naphthoflavone (0, 1, 2, 5, 10, and 20 nM) was added to the reaction mixtures, each containing varied concentration of phenacetin (20, 50, and 100 μM). The other conditions were similar to those used in the P450 inhibition study, as described earlier.

### 2.6. Inhibitory Effects of Obtusifolin against Human Cytochrome P450s Activity

The inhibitory potential of obtusifolin against the biotransformation of eight P450 probe substrates was examined using previously reported methods with slight changes [21,22]. The following P450 isoform-specific substrates were used: coumarin (CYP2A6); bupropion (CYP2B6); amodiaquine (CYP2C8); tolbutamide (CYP2C9); omeprazole (CYP2C19); dextromethorphan (CYP2D6); chlorzoxazone (CYP2E1); and midazolam (CYP3A). Mixtures of pooled HLM, P450 substrates, and obtusifolin (0, 0.05, 0.2, 0.5, 2, 5, or 10 µM for CYP1A2; and 0, 0.5, 2, 5, 20, or 50 µM for the other P450 isoforms) were pre-incubated (5 min, 37 °C). NGS was introduced to start the reaction, which was followed by an additional incubation (15 min). Following reaction termination, aliquots of the supernatants were analyzed with LC-MS/MS.

### 2.7. Inhibitory Effects of Obtusifolin on Human Recombinant CYP1A1 and CYP1A2 Enzymes

The inhibitory effects of obtusifolin on the CYP1A-mediated ethoxyresorufin *O*-deethylation (EROD) activity of rCYP1A1 and rCYP1A2 enzymes (10 pmol/mL) were investigated. Obtusifolin (0, 0.05, 0.2, 0.5, 2, 5, or 10 µM) was added to the reaction mixture containing 7-ethoxyresorufin (2 μM). The other conditions, except for the incubation time (20 min), were similar to those used in the P450 inhibition study, as described earlier.

### 2.8. LC-MS/MS Analysis

Reverse phase column (Kinetex XB-C18, 100 × 2.10 mm; Phenomenex, Torrance, CA, USA) and triple-quadrupole mass spectrometer (LCMS 8060, Shimadzu, Kyoto, Japan) with an ultra high-performance LC system (Nexera X2, Shimadzu) analyze all the metabolites. Mobile phases were 0.1% formic acid in water (A) and 0.1% formic acid in acetonitrile (B). The elution condition was as follows: 0–0.5 min (B: 8%), 0.5–5 min (B: 8%→60%), 5–6 min (B: 60%), 6–6.1 min (B: 60%→8%), and 6.1–10 min (B: 8%) for the analysis of metabolites of the nine P450 substrates; and 0–1 min (B: 5%), 1–5 min (B: 5%→90%), and 5.1–7 min (B: 90%→5%) for the analysis of resorufin. Electrospray ionization was performed in the positive- and negative-ion modes at 4.0 and −3.5 kV, respectively. For each metabolite, quantitation was carried out in selected reaction monitoring (SRM) mode using the following precursor-to-product ion transition: *m*/*z* 152 > 110 for acetaminophen (CYP1A2), *m*/*z* 214 > 186 for resorufin (CYP1A1 and CYP1A2), *m*/*z* 163 > 107 for 7-hydroxycoumarin (CYP2A6), *m*/*z* 256 > 238 for 6-hydroxybupropion (CYP2B6), *m*/*z* 328 > 283 for *N*-desethylamodiaquine (CYP2C8), *m*/*z* 287 > 89 for 5-hydroxytolbutamide (CYP2C9), *m*/*z* 362 > 214 for 4-hydroxyomeprazole (CYP2C9), *m*/*z* 258 > 157 for dextrorphan (CYP2D6), *m*/*z* 184 > 120 for 6-hydroxychlorzoxazone (CYP2E1), *m*/*z* 342 > 203 for 1′-hydroxymidazolam (CYP3A), and *m*/*z* 295 > 100 for trimipramine (IS) [23,24].

### 2.9. Data Analysis

WinNonlin software (Pharsight, Mountain View, CA, USA) was used to calculate IC_50_ values. Based on Lineweaver–Burk double reciprocal plots, secondary plots of Lineweaver–Burk plots versus obtusifolin concentrations, and visual inspection of Dixon plots, the type of inhibition and apparent kinetic parameters for inhibitory activity (*K*_i_) were estimated.

## 3. Results and Discussion

### 3.1. Inhibition of CYP1A2 Activity by Five Major Phytochemicals from Cassiae Semen

**Figure 2:**
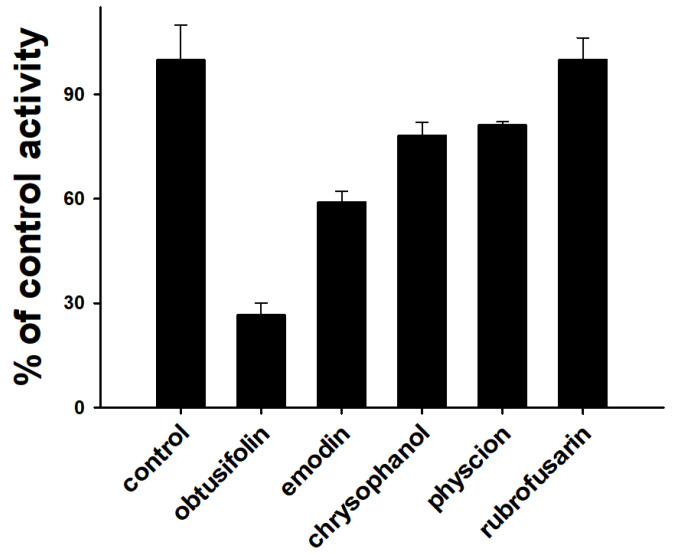
Inhibitory effects of obtusifolin, emodin, chrysophanol, physcion, and rubrofusarin on CYP1A2-mediated phenacetin O-deethylation in human liver microsomes. Pooled human liver microsomes (0.25 mg/mL) were incubated with phenacetin (100 µM) in the presence or absence of each chemical (0.5 µM) at 37 °C for 15 min. The activity is expressed as the percentage of the control activity. The data are depicted as the average obtained from triplicate experiments (*n* = 3).

In the previous study, Tang et al. (2009) reported that rhein, an anthraquinone in rhubarb root, inhibited CYP1A2-catalyzed POD activity with a *K*_i_ value of 62 µM in rat liver microsomes [25]. Liu et al. (2021) also demonstrated that emodin more selectively inhibited CYP1A2-mediated POD activity (IC_50_ = 0.5 µM) than the activities of other P450 isoforms (IC_50_ > 9.6 µM) in HLMs [8]. Based on these data, we explored the possibility of CYP1A2 inhibition by four anthraquinones (chrysophanol, emodin, obtusifolin, and physcion) and one naphthopyrone (rubrofusarin), which were isolated from *Cassiae* semen in HLMs (Figure 1). The amount of acetaminophen selectively produced by CYP1A2 was measured using LC-MS/MS in SRM mode and determined by the area ratio (acetaminophen/IS area) to quantify CYP1A2-mediated POD activity (Figure S1). Anthraquinones (IC_50_ < 2.2 µM) more strongly inhibited CYP1A2 activity than naphthopyrone (IC_50_ > 6 µM). Among the four anthraquinones, obtusifolin (IC_50_ = 0.19 µM) was found to be the strongest inhibitor of CYP1A2 activity (Table 1 and Figure 2). The inhibitory potential of obtusifolin was higher than those of furafylline (IC_50_ = 1.56 µM), isopimpinellin (IC_50_ = 0.46 µM), and trioxsalen (IC_50_ = 0.79 µM) [8,26]. The inhibitory potential (IC_50_ = 0.79 µM) of emodin against CYP1A2 in HLMs was observed to be similar to the previously reported value (IC_50_ = 0.50 µM) [8]. The inhibitory potentials of chrysophanol (IC_50_ = 1.99 µM) and physcion (IC_50_ = 2.16 µM) against CYP1A2 in HLMs were higher than those observed against rCYP1A2 (IC_50_ = 0.29 and 0.88 µM, respectively) [16]. This could be attributed to the differences in incubation conditions, such as enzyme source (HLM versus rCYP1A2) or CYP1A2 probe substrate (phenacetin versus melatonin) [16,23]. For example, the IC_50_ value of mollugin for CYP1A2-mediated POD activity in rCYP1A2 was five-fold higher than that in HLMs [27].

### 3.2. Binding Modes of Obtusifolin Assessed by Molecular Docking Simulation

**Figure 3:**
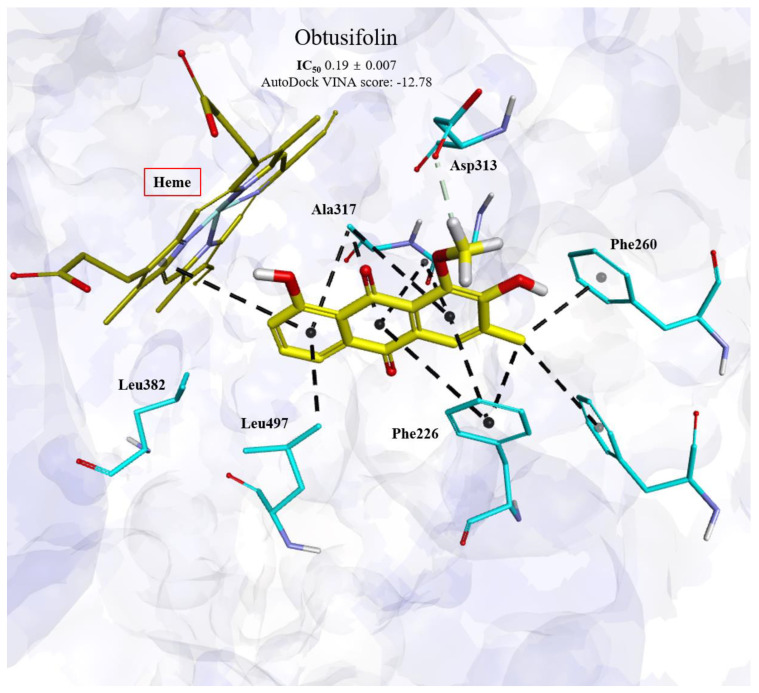
Molecular docking pose of obtusifolin in CYP1A2 (PDB ID: 2HI4). The carbon atoms of the interacting residues in CYP1A2 are displayed as cyan sticks and labeled. Heme is represented by red sticks. The carbon atoms in the ligand are shown as yellow sticks. The non-classified hydrogen bond and hydrophobic interactions are depicted by light green and black dotted lines, respectively. Leu382 is highlighted to demonstrate that steric bump formation with the ligand is not possible (Please find more details in the Discussion section).

To explore the critical interactions between anthraquinones from *Cassiae* semen and human CYP1A2 from the perspective of protein–ligand interactions and to clarify the mechanism behind the enhanced CYP1A2 inhibitory activity of obtusifolin compared with other anthraquinones, molecular docking simulations were performed using a previously reported crystal structure of CYP1A2 (PDB ID: 2HI4) [19]. As shown in Figure 3, anthraquinones could be readily docked into the active site of the CYP1A2 enzyme, while the binding area in the active site was highly overlapped with that of phenacetin (PDB accession number: 3EBS), implying that anthraquinones could occupy the binding area of the CYP1A2 substrate and thereby serving as inhibitors of CYP1A2 enzyme.

The potential critical interactions between anthraquinones and CYP1A2 enzyme were comprehensively analyzed using the docking complex model. Obtusifolin binds to the CYP1A2 active site with the lowest docking score of −12.78 kcal/mol (Table 2). As shown in Figure 3, obtusifolin hydrophobically interacts mainly with heme, Phe226, Phe256, Phe260, Gly316, Ala317, and Leu497 in the catalytic site of CYP1A2. It also forms a nonclassical hydrogen bond with Asp313 through its methoxy group. The complex structure of CYP1A2 and α-naphthoflavone exhibited similar hydrophobic contacts with Phe226, Gly316, Ala317, and Leu497; thus, these interactions may be conserved for CYP1A2 inhibition.

Conversely, emodin was the second-best CYP1A2 inhibitor among the examined compounds. Its hydroxyl group interaction with heme, along with hydrophobic contacts, contributed to a high CYP1A2 inhibitory activity (Figure S3). Chrysophanol harbors a methyl group in the same location as that in obtusifolin. However, it lacks a hydroxyl group that would facilitate its interaction with heme like emodin. (Figure S4). Rubrofusarin, the least potent inhibitor, likely has steric hindrance with Leu382, as its methoxy group is within 1 Å of this amino acid (Figure S5). Thus, the binding orientation of rubrofusarin might be altered owing to these differences from obtusifolin, thereby reducing its CYP1A2 inhibitory capacity. The bulky methoxy group in physcion, the second least effective inhibitor, might have lessened its inhibitory action on CYP1A2; however, the lack of a hydroxyl group, as in rubrofusarin, might have contributed to its superior CYP1A2 inhibitory function compared with that of rubrofusarin (Figure S6).

As observed in rubrofusarin and physcion, the presence of bulky methoxy groups in disadvantageous positions in the molecules may result in steric hindrance with Leu382, which could result in inefficient CYP1A2 inhibition (Figures S5 and S6). However, as observed in emodin, small functional groups, such as the hydroxyl group, may result in moderate action against CYP1A2. The presence of a methyl group (equivalent to the 14th atomic position in obtusifolin) in the molecules may result in moderate inhibitory activity, as seen in the case of emodin and chrysophanol, if the methoxy group is absent in both the favorable and unfavorable locations of the molecules (Figures S3 and S4).

In summary, the docking models suggest that the high inhibitory activity of obtusifolin against CYP1A2 primarily results due to the combination of extensive hydrophobic interactions with three Phe residues (Phe226, Phe256, and Phe260) via its methyl group and the non-classical hydrogen bond with Asp313 via the methoxy group (Figure 3). The methoxy group at this particular position in obtusifolin is not present in other molecules, suggesting its significant role in the effective inhibition of CYP1A2 (Figure 3).

### 3.3. Characterizatin of Inhibition Kinetics of Obtusifolin against CYP1A2

**Figure 4:**
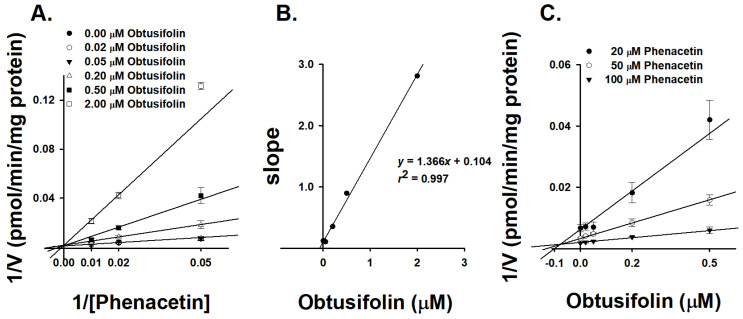
Representative Lineweaver–Burk (**A**), secondary Lineweaver–Burk (**B**), and Dixon plots (**C**) obtained from the inhibition kinetics of CYP1A2-mediated phenacetin *O*-deethylation in the presence of different concentrations of obtusifolin in human liver microsomes (HLMs). An increasing concentration of phenacetin (20, 50, and 100 µM) was incubated with HLMs (0.25 mg/mL) and an NADPH generating system at 37 °C for 15 min in the presence or absence of obtusifolin. The data are depicted as the average obtained from triplicate experiments (*n* = 3).

Obtusifolin inhibited microsomal CYP1A2 activity with IC_50_ values of 0.19 μM, therefore, we sought to clarify the underlying mechanism of this inhibition. We measured the inhibition constant of α-naphthoflavone, a well-known CYP1A2 selective inhibitor, to validate our experimental system. α-Naphthoflavone inhibited CYP1A2-mediated POD activity with a *K*_i_ value of 0.0075 μM in HLMs. Our results with α-naphthoflavone are consistent with that of a previous study, which reported potent inhibition of CYP1A2 activity in HLMs by α-naphthoflavone with a *K*_i_ value of 0.01 μM using phenacetin as the CYP1A2 probe substrate [28]. This highlighted the suitability of our experimental system for evaluating the inhibitory ability of obtusifolin against CYP1A2. According to the Lineweaver–Burk plot (Figure 4A), obtusifolin presented a typical pattern of competitive inhibition for CYP1A2-mediated POD activity in HLMs, with a *K*_i_ value of 0.11 μM (Table 3). The secondary Lineweaver–Burk plot also demonstrated a linear correlation (Figure 4B, *R^2^* = 0.997). The Dixon plot intersected above the *X*-axis, indicating that obtusifolin inhibited CYP1A2 in a competitive manner (Figure 4C) [29]. Obtusifolin exhibited a stronger inhibitory potency than machilin A (*K*_i_ = 0.71 μM) [30], isopimpinellin (*K*_i_ = 1.2 μM) [31], and mollugin (*K*_i_ = 3.74 μM) [27], but was less potent than α-naphthoflavone (*K*_i_ = 0.01 μM), a well-known strong selective inhibitor of CYP1A2 [26]. Obtusifolin also inhibited CYP1A2 activity in the rCYP1A2 isoform with a *K*_i_ value of 0.21 μM, which was similar to the value observed in HLMs (Table 3).

In addition, several CYP1A2 inhibitors, including antofloxacin [26], furafylline [32] and isopimpinellin [31], are time-dependent inhibitors of CYP1A2. We investigated the effect of incubation time on the IC_50_ values of obtusifolin against CYP1A2. We found that obtusifolin showed time-independent inhibition of CYP1A2-mediated POD activity with an IC_50_ shift value of 1.46 (IC_50_ values of 0.13 and 1.19 µM, with and without NGS preincubation, respectively). A chemical with an IC_50_ fold-shift decrease of <1.50 is considered a time-independent inhibitor [33]. α-Naphthoflavone, a time-independent inhibitor [34], also exhibited an IC_50_ shift value of 0.83.

In this study, we used in vitro experimental system such as human liver microsomes and recombinant P450 isoforms for enzyme inhibition studies. Since these systems have a limitation in that they can not reflect the exact intracellular concentration of the test compounds, it will be necessary to conduct enzyme inhibition studies using cultured cells, such as human hepatocytes.

### 3.4. Selective Inhibition of CYP1A2 Activity by Obtusifolin

**Figure 5:**
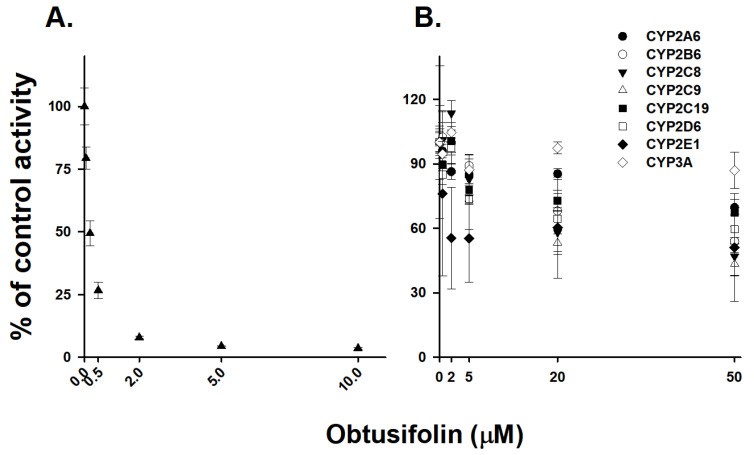
Inhibitory effects of obtusifolin on (**A**) phenacetin *O*-deethylation (CYP1A2, ▲) and (**B**) coumarin 7-hydroxylation (CYP2A6, ●), bupropion 6-hydroxylation (CYP2B6, ○), amodiaquine N-deethylation (CYP2C8, ▼), tolbutamide 4-hydroxylation (CYP2C9, △), omeprazole 4-hydroxylation (CYP2C19, ■), dextromethorphan *O*-demethylation (CYP2D6, □), chlorzoxazone 6-hydroxylation (CYP2E1, ◆), and midazolam 1′-hydroxylation (CYP3A, ◇) during incubation with human liver microsomes (0.25 mg/mL). The activity was expressed as the percentage of the control activity. The data are represented as the mean ± S.D. (*n* = 3).

**Figure 6:**
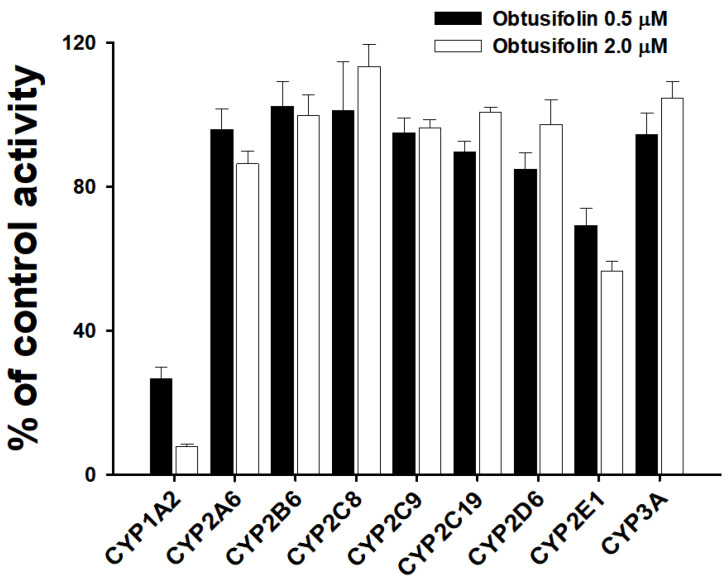
Inhibitory effects of obtusifolin [0.5 (■) and 2.0 µM (□)] on enzyme activities of nine P450 isoforms in human liver microsomes (0.25 mg/mL). The data are depicted as the average obtained from triplicate experiments (*n* = 3).

Obtusifolin had a potent inhibitory effect on CYP1A2-mediated phenacetin *O*-deethylation (IC_50_ = 0.19 µM). To evaluate whether obtusifolin selectively inhibits CYP1A2, we investigated its inhibitory activity against eight other P450 isoforms. Obtusifolin showed weak inhibition of CYP2C8 and CYP2C9, with IC_50_ values of 31.4 and 28.6 µM, respectively, and negligible inhibition (IC_50_ > 50 µM) of CYP2A6, CYP2B6, CYP2C19, CYP2D6, CYP2E1, and CYP3A activities (Table 4 and Figure 5). Obtusifolin exhibited over 150-fold selectivity (CYP1A2 vs. other P450s), whereas HYIpro-3-1 [35] and mollugin [27] showed 125- and 25-fold selectivity for CYP1A2 inhibition, respectively. At a 0.5 µM obtusifolin concentration, which is approximately seven-fold greater than the *K*_i_ value, obtusifolin was observed to inhibit CYP1A2 activity by 73.3% and only slightly affected the enzyme activities of the other eight P450s. Obtusifolin weakly inhibited CYP2E1-mediated chlorzoxazone hydroxylase activity (30.7%) at 0.5 µM concentration (Figure 6). Similar to α-naphthoflavone and furafylline, obtusifolin could also potentially be used as a potent and selective CYP1A2 inhibitor in drug metabolism studies.

### 3.5. Chemopreventive Effects of Obtusifolin

**Figure 7:**
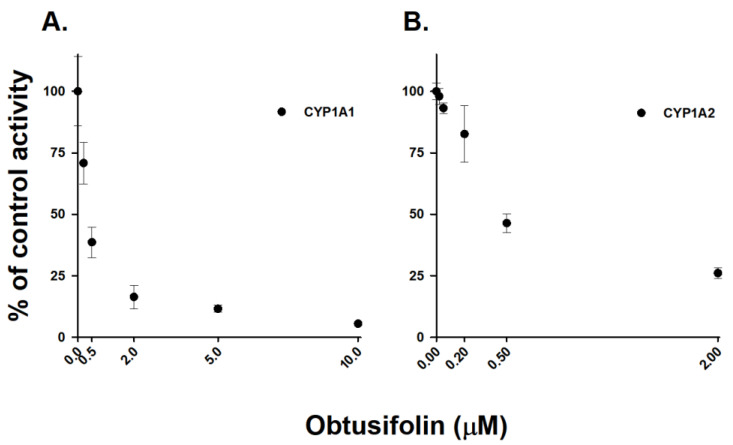
Inhibitory effects of obtusifolin on CYP1A1- and CYP1A2-mediated ethoxyresorufin *O*-deethylation in recombinant human CYP1A1 (**A**) and CYP1A2 (**B**) enzymes. Recombinant human P450s (10 pmol/mL) were incubated with ethoxyresorufin (2 µM) in the presence or absence of obtusifolin (0–10 µM) at 37 °C for 20 min. The activity is expressed as the percentage of the control activity. The data are depicted as the average obtained from triplicate experiments (*n* = 3).

The CYP1A family plays a vital role in the metabolic activation of carcinogens, including mycotoxins, such as aflatoxin B1 [11], and polycyclic aromatic hydrocarbons, such as benzo[a]pyrene [12]. The most reactive metabolite, benzo[a]pyrene-7,8-diol-9,10-epoxide, which is responsible for tumor production in new born mice, is produced by CYP1A enzymes [36]. Therefore, the selective inhibition of CYP1A-mediated activation of procarcinogens is potentially a crucial chemopreventive strategy. Sulforaphane [14], resveratrol [13], and curcumin [37] are potent chemopreventive agents that directly inhibit CYP1A enzymes. The CYP1A family contains only two functional isoforms, namely CYP1A1 and CYP1A2. We evaluated it inhibitory potential against the CYP1A family to confirm the potential of obtusifolin as a chemopreventive compound. Obtusifolin was incubated with human rCYP1A1 and rCYP1A2 isoforms using 7-ethoxyresorufin as the substrate. Obtusifolin similarly inhibited the CYP1A1- and CYP1A2-mediated EROD activities with IC_50_ values of 0.39 and 0.57 µM, respectively (Figure 7), indicating that obtusifolin can be considered a chemopreventive agent. The inhibitory potential of obtusifolin against CYP1A1 isoform was higher than those of resveratrol (IC_50_ = 23 µM) [13] and curcumin (IC_50_ = 0.74 µM) [38], which are well-known chemopreventive CYP1A inhibitors. However, it was less potent than α-naphthoflavone (IC_50_ = 0.06 µM), a well-known strong inhibitor of CYP1A1 [39]. In addition, in terms of CYP1A-mediated POD activity, obtusifolin exhibited over six-fold selectivity for rCYP1A1 (IC_50_ = 0.06 µM) over rCYP1A2 (IC_50_ = 0.37 µM) (Table 5). Its selectivity for CYP1A1 inhibition over CYP1A2 is similar to that of 7-hydroxyflavone (six-fold) [40]; however, it was lower than resveratrol which demonstrated 51-fold selectivity [13]. Based on this obtusifolin-mediated selective and potent inhibition of CYP1A1 and CYP1A2 enzymes, the in vivo chemopreventive effects of obtusifolin should be evaluated in the future.

### 3.6. Evaluation of Drug Interaction Potential of Obtusifolin

It was estimated that an in vivo interaction potential via the inhibition of P450 would likely occur if the ratio of inhibitor C_max_/*K*_i_ exceeded 1.0, and would be possible if it was between 0.1 and 1.0 [18]. Based on obtusifolin’s maximum concentrations (0.86 and 0.54 μM) in rat blood after a single oral administration of Semen Cassiae extracts (1.25 g/kg; contents: 5.01 mg/g obtusifolin) [41] and obtusifolin (1.3 mg/kg) [42], the respective values of C_max_/*K*_i_ were 7.82 and 4.91 from the data of pHLMs (*K*_i_ = 0.11 μM), suggesting that obtusifolin has possible drug interaction potential with CYP1A2 substrate drugs. Thus far, there have been no reports on the pharmacokinetics of obtusifolin in humans, therefore, it is difficult to estimate the drug interaction potential of obtusifolin for humans. However, rhein, which is one of the anthraquinone compounds like obtusifolin, has been reported to reach a maximum plasma concentration of 9.52 and 18.8 μM after oral dose of 50 and 100 mg, respectively, in humans [43]. Therefore, obtusifolin might have drug interactions with CYP1A2 substrate drugs, such as imipramine [44], olanzapine [45], and tizanidine [46]. Therefore, in vivo studies are necessary to determine whether drug interactions between obtusifolin and CYP1A2 substrates have clinical relevance.

## 4. Conclusions

In this study, we investigated the inhibitory potential of four anthraquinones and one naphthopyrone isolated from *Cassiae* semen on the activity of CYP1A2 isoform in HLMs. Among five compounds tested, obtusifolin potently inhibited CYP1A2-mediated POD activity, with *K*_i_ values lower than 0.5 µM in HLMs and rCYP1A2. Furthermore, obtusifolin selectively inhibited CYP1A1 and CYP1A2 enzymes; however, it had negligible inhibitory effects on other P450 isoforms, highlighting its potential chemopreventive effects. In conclusion, we confirmed the selective and potent inhibitory effects of obtusifolin against CYP1A enzymes in HLMs and recombinant CYP1A enzymes.

## Figures and Tables

**Table 1:** Inhibitory effects of four anthraquinones (obtusifolin, emodin, chrysophanol, and physcion) and one naphthopyrone (rubrofusarin) against CYP1A2-mediated phenacetin *O*-deethylase activity in human liver microsomes.

Enzyme	IC_50_ (µM) ^(1)^				
CYP1A2	Obtusifolin	Emodin	Chrysophanol	Physcion	Rubrofusarin
	0.19 ± 0.01	0.79 ± 0.26	1.99 ± 0.23	2.16 ± 0.48	6.33 ± 1.38

^(1)^ Values represent the average ± standard error of triplicate.

**Table 2:** Interactions and binding energy of four anthraquinones (obtusifolin, emodin, chrysophanol, and physcion) and one naphthopyrone (rubrofusarin) with CYP1A2 isoform.

Chemical	IC_50_ (µM)	Docking Score (kcal/mol)	Interactions
Obtusifolin	0.19 ± 0.01	−12.78	Phe226, Phe256, Phe260, Ala317, Asp313, and Leu497
Emodin	0.79 ± 0.26	−11.38	Phe226, Phe260, Ala317, and Leu497
Chrysophanol	1.99 ± 0.23	−11.66	Phe226, Phe260, Ala317, and Leu497
Physcion	2.16 ± 0.48	−8.34	Phe226, Phe260, Ala317, and Leu497
Rubrofusarin	6.33 ± 1.38	−7.56	Thr124, Phe226, Phe256, Phe260, Ala317, and Leu497

**Table 3:** Enzyme inhibition constants (*K*_i_ values) for the inhibition of CYP1A2-mediated phenacetin *O*-deethylation in human liver microsomes (HLMs) or recombinant CYP1A2 isoform by obtusifolin and α-naphthoflavone.

Inhibitor	HLM/rCYP1A2	IC_50_ (μM)	*K*_i_ (μM)
Obtusifolin	HLMs	0.19 ± 0.01	0.11 ± 0.02
	rCYP1A2	0.37 ± 0.11	0.21 ± 0.053
α-Naphthoflavone	HLMs	0.0061 ± 0.0008	0.0075 ± 0.0010

**Table 4:** Inhibitory effects of obtusifolin against nine cytochrome P450 isoforms in human liver microsomes.

P450 Isoforms	IC_50_ (µM)
CYP1A2	0.19 ± 0.01
CYP2A6	>50
CYP2B6	>50
CYP2C8	31.44 ± 9.85
CYP2C9	28.64 ± 6.98
CYP2C19	>50
CYP2D6	>50
CYP2E1	>50
CYP3A	>50

**Table 5:** Inhibitory effects (IC_50_) of obtusifolin against recombinant CYP1A1 and CYP1A2.

Recombinant Cytochrome P450	Substrate	IC_50_ (μM)
rCYP1A1	Phenacetin	0.06 ± 0.02
	7-Ethoxyresorufin	0.39 ± 0.06
	9-cis-Retinal	>10
rCYP1A2	Phenacetin	0.37 ± 0.11
	7-Ethoxyresorufin	0.57 ± 0.11
	9-cis-Retinal	0.15 ± 0.03

## Data Availability

All data in this study have been included in this manuscript.

## Supplementary Materials

The following supporting information can be downloaded at: https://www.mdpi.com/article/10.3390/pharmaceutics14122683/s1, Figure S1: Selected reaction chromatograms for acetaminophen obtained from an acetaminophen standard (A) 200 µM and an incubation study in human liver microsomes (B) 0 µM obtusifolin; (C) 0.5 µM obtusifolin; Figure S2: Molecular docking pose of α-naphthoflavone in CYP1A2; Figure S3: Molecular docking pose of emodin in CYP1A2; Figure S4: Molecular docking pose of chrysophanol in CYP1A2; Figure S5: Molecular docking pose of physcion in CYP1A2; Figure S6: Molecular docking pose of rubrofusarin in CYP1A2.
