# Supplementary material for: Potent and Selective Inhibition of CYP1A2 Enzyme by Obtusifolin and Its Chemopreventive Effects
Source: Pharmaceutics. 2022 Dec 1;14(12):2683. doi: 10.3390/pharmaceutics14122683 (PMC9786103; doi:10.3390/pharmaceutics14122683)
Supplement: Supplementary file 1 [file pharmaceutics-14-02683-s001.zip › pharmaceutics-2033536-supplementary.pdf]

SUPPLEMENTARY DATA

Potent and selective inhibition of CYP1A2 enzyme by obtusifolin and its chemopreventive effects

Eun-Ji Park [1,†], Keunwan Park [2,†], Prasannavenkatesh Durai [2], Ki-Young Kim [1], So-Young Park [1], Jaeyoung Kwon [2], Hee Ju Lee [2], Cheol-Ho Pan [2,*] and Kwang-Hyeon Liu [1,3,*]

[1] BK21 FOUR KNU Community-Based Intelligent Novel Drug Discovery Education Unit, College of Pharmacy and Research Institute of Pharmaceutical Sciences, Kyungpook National University, Daegu 41566, Republic of Korea

[2] Mass Spectrometry Based Convergence Research Institute, Kyungpook National University, Daegu 41566, Republic of Korea

[3] Natural Product Informatics Research Center, KIST Gangneung Institute of Natural Products, Gangneung 25451, Republic of Korea

* Correspondence: panc@kist.re.kr (C.-H.P); dstlkh@knu.ac.kr (K.-H.L.); Tel.: +82-33-650-3652 (C.-H.P.); +82-53-950-8567 (K.-H.L.); Fax: +82-33-650-3419 (C.-H.P.); +82-53-950-8557 (K.-H.L.);

† These authors contributed equally to this work.

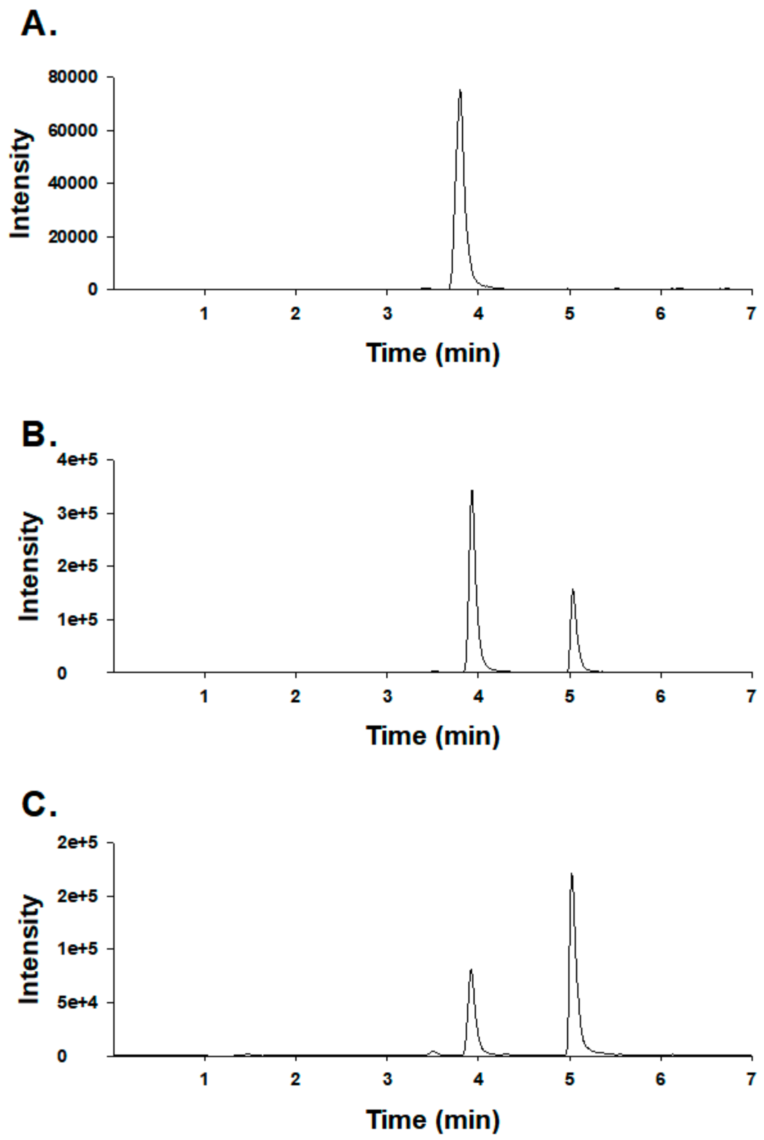

Figure S1. Selected reaction chromatograms for acetaminophen obtained from an acetaminophen standard (A) 200 μM and an incubation study in human liver microsomes (B) 0 μM obtusifolin; (C) 0.5 μM obtusifolin.

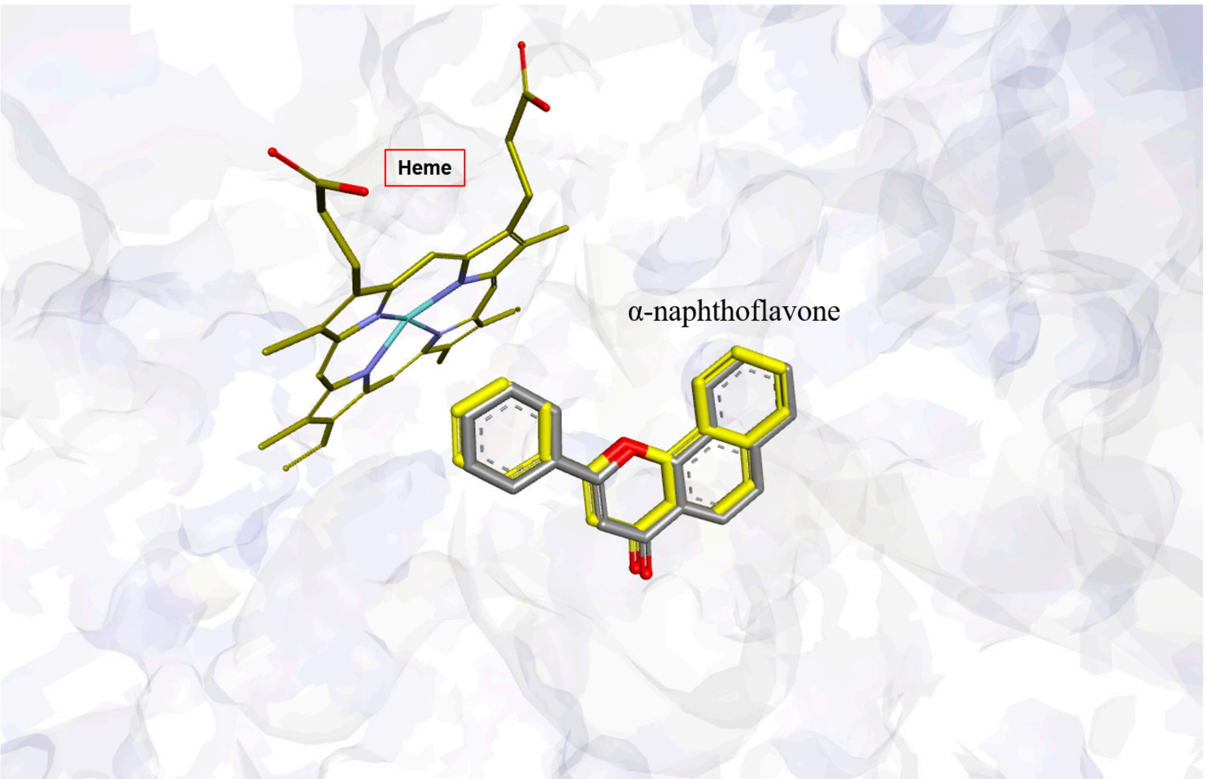

Figure S2. Molecular docking pose of α-naphthoflavone in CYP1A2.

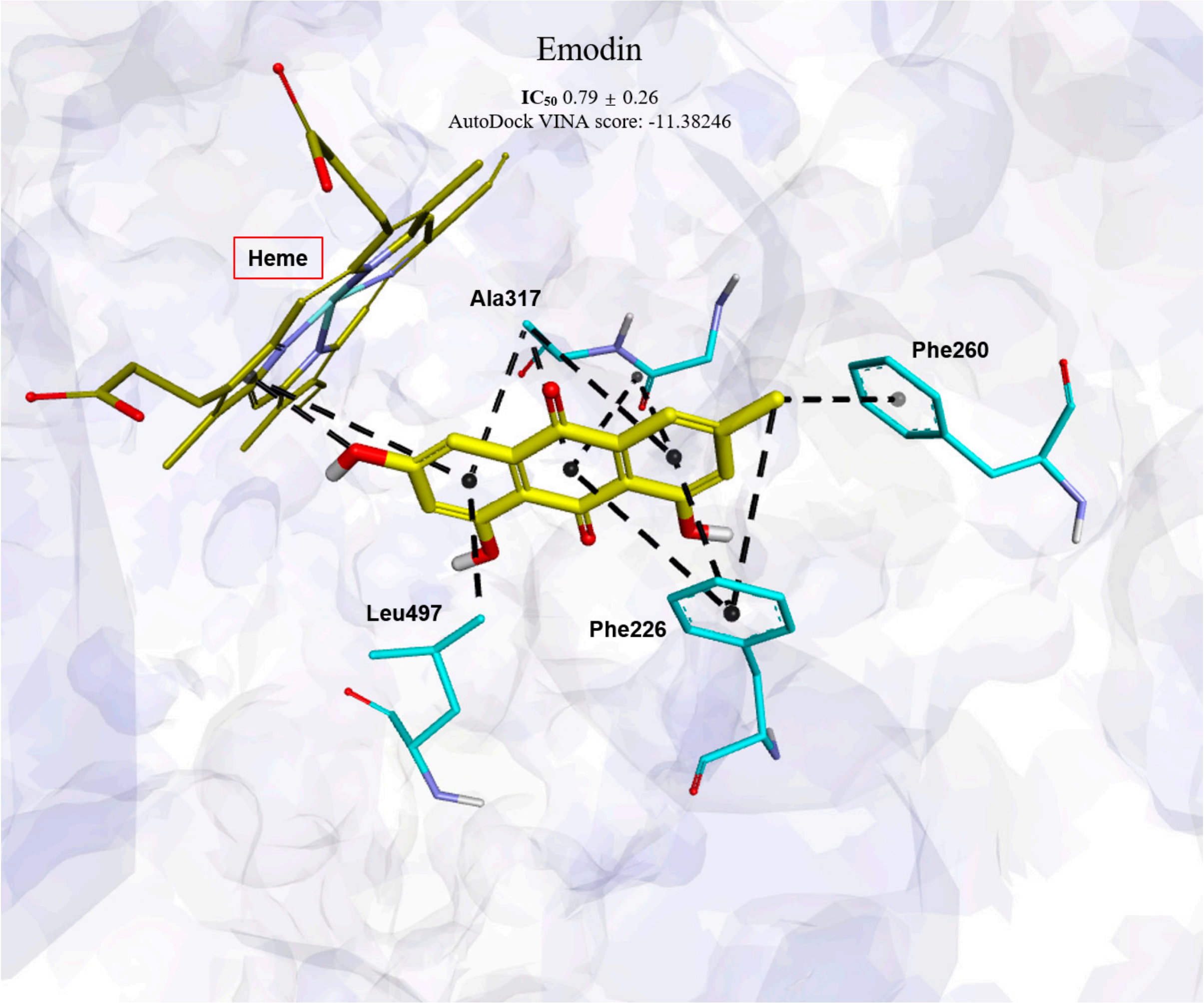

Figure S3. Molecular docking pose of emodin in CYP1A2.

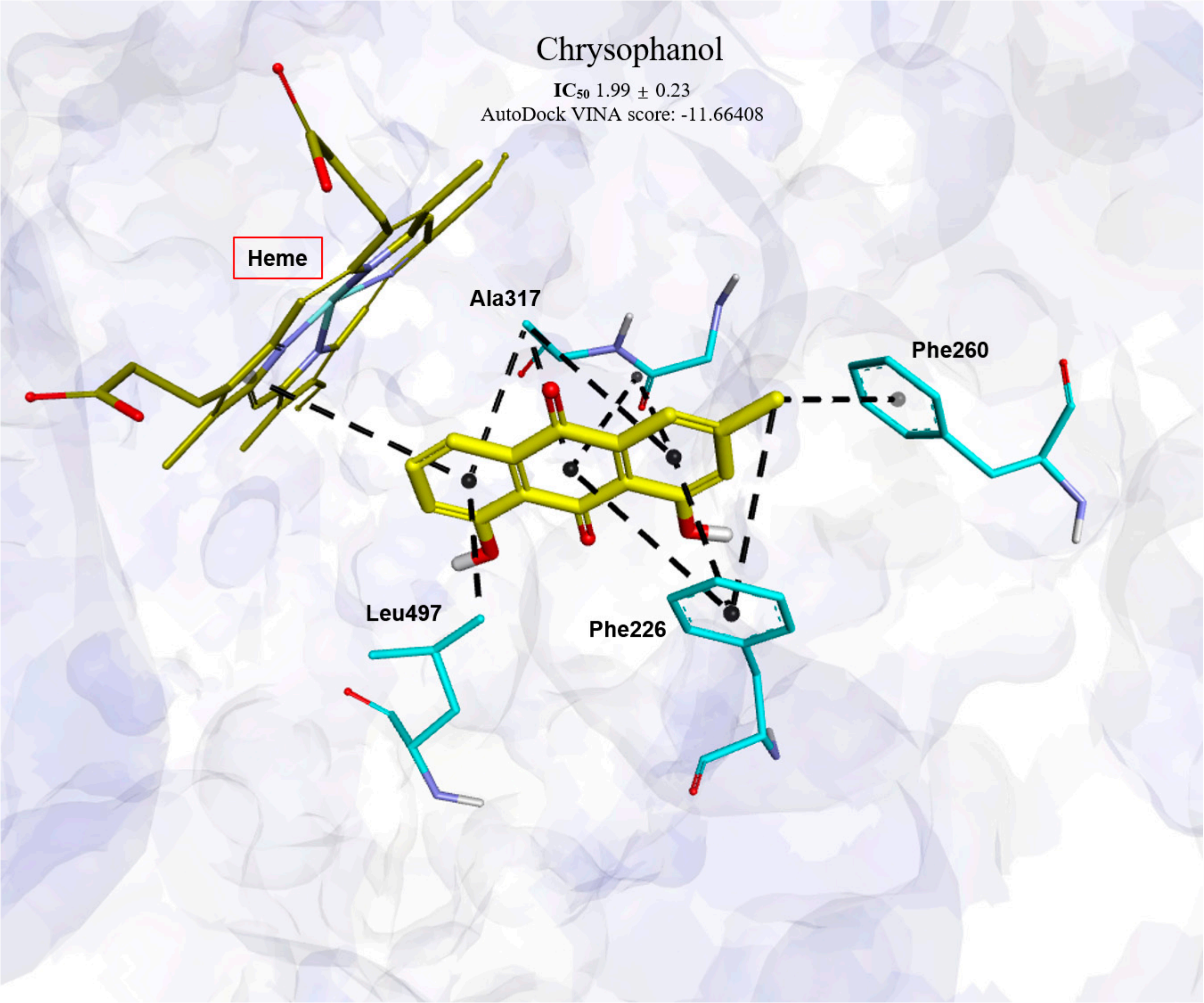

Figure S4. Molecular docking pose of chrysophanol in CYP1A2.

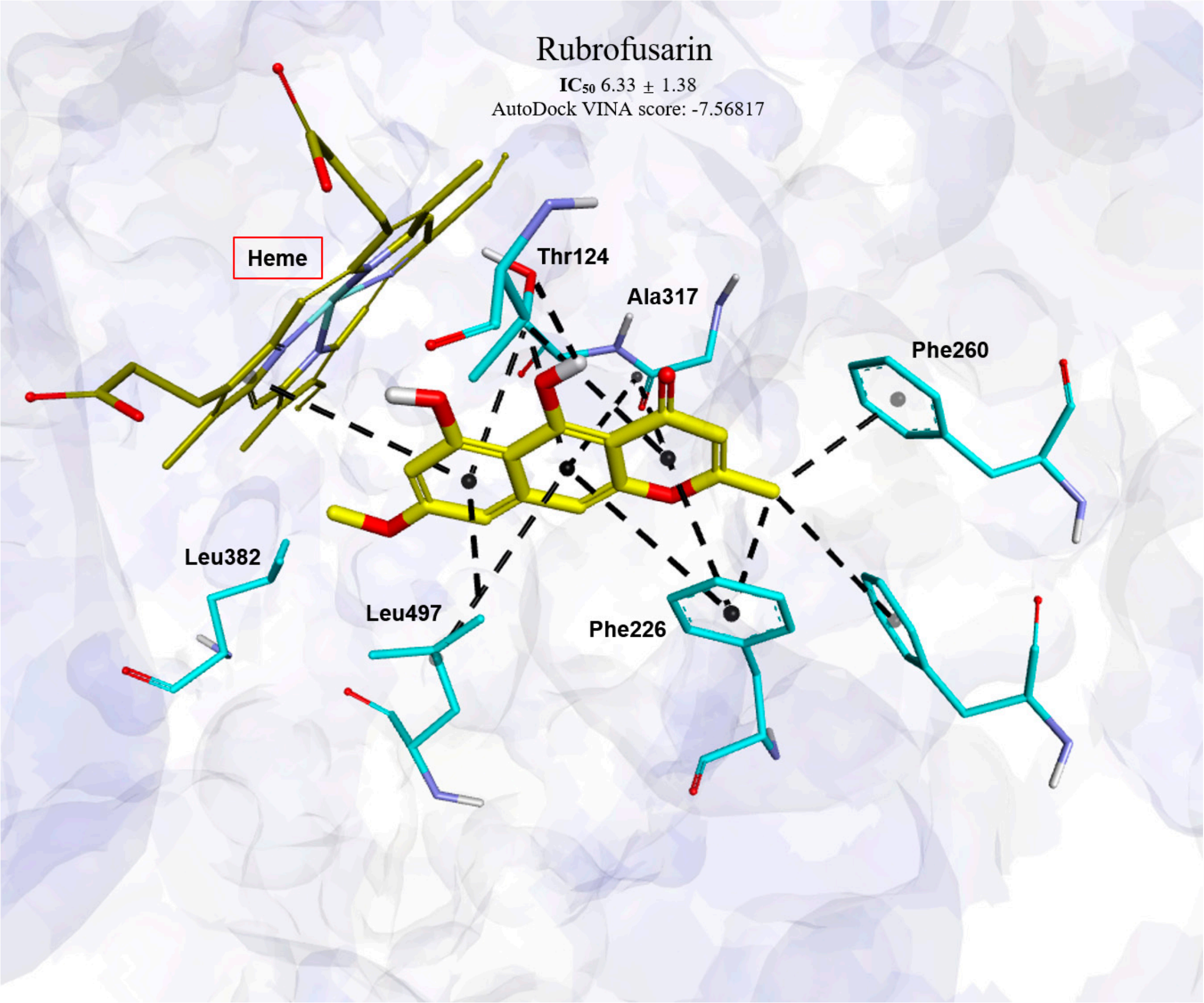

Figure S5. Molecular docking pose of rubrofusarin in CYP1A2.

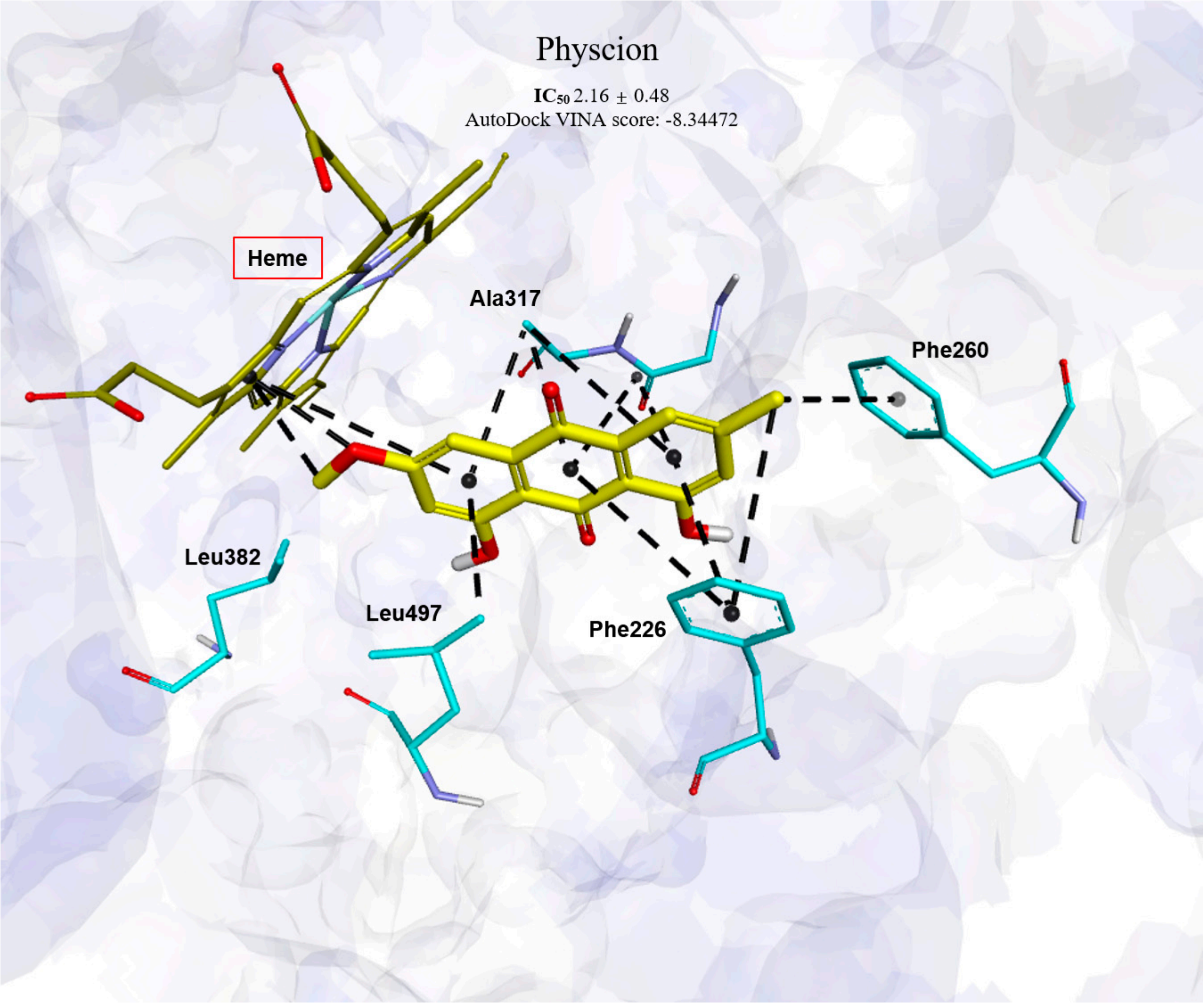

Figure S6. Molecular docking pose of physcion in CYP1A2.
